# Chemical, Physicochemical and Sensorial Characterization of Nitrite-Free Dry-Cured Bísaro Shoulders

**DOI:** 10.3390/foods11193079

**Published:** 2022-10-04

**Authors:** Ana Leite, Lia Vasconcelos, Iasmin Ferreira, Ainhoa Sarmiento-García, Rubén Domínguez, Eva María Santos, Paulo C. B. Campagnol, Sandra Rodrigues, José M. Lorenzo, Alfredo Teixeira

**Affiliations:** 1 Centro de Investigação de Montanha (CIMO), Instituto Politécnico de Bragança, Campus de Santa Apolónia, 5300-253 Bragança, Portugal; 2 Laboratório para a Sustentabilidade e Tecnologia em Regiões de Montanha, Instituto Politécnico de Bragança, Campus de Santa Apolónia, 5300-253 Bragança, Portugal; 3 Área de Tecnoloxía dos Alimentos, Facultade de Ciencias de Ourense, Universidade de Vigo, 32004 Ourense, Spain; 4 Área de Producción Animal, Department of Construcción y Agronomía, Facultad de Ciencias Agrarias y Ambientales, Universidad de Salamanca, 37007 Salamanca, Spain; 5 Centro Tecnológico de la Carne de Galicia, Avd. Galicia N° 4, Parque Tecnológico de Galicia, San Cibrao das Viñas, 32900 Ourense, Spain; 6 Área Académica de Química, Universidad Autónoma del Estado de Hidalgo, Mineral, Pachuca 42183 , Mexico; 7 Departmento de Tecnologia e Ciência de Alimentos, Universidade Federal de Santa Maria, Santa Maria 97105-900, Brazil

**Keywords:** Bísaro breed, meat products, fatty acids, meat quality, nitrite-free product, panelists

## Abstract

The aim of the current experiment was to characterize and evaluate the effect of the dry-curing process on chemical composition, physicochemical properties, and sensory characteristics of the dry-cured Bísaro shoulders. For this purpose, thirty-eight raw forelegs were used, and no nitrites were added during the dry-curing process. This process increased protein, fat, ash content, and pH, with a decrease in moisture and water activity (*p* < 0.001). The dry-cured shoulders were darker (L*), less red (a*), and less yellow (b*) than the raw shoulders (*p* < 0.001), and this may be mainly due to the moisture reduction. The proportion of polyunsaturated fatty acids (PUFA) decreased during processing, whereas the saturated fatty acids (SFA) and monounsaturated fatty acids (MUFA) increased (*p* < 0.001), which could be related with the oxidative degradation. The sensory analysis showed that dry-cured Bísaro shoulders presented similar organoleptic characteristics to other dry-cured meat products. Also, the chemical composition and fatty acid profile of the dry-cured Bísaro shoulder showed results comparable to those of other cured products. This study revealed that it is possible to obtain safer and healthier dry-cured Bísaro shoulder products judging by these characteristics, since nitrites were not added in its preparation. These findings, along with the product’s high sensory attributes similar to more popular products such as ham, would give more advantage for its acceptability and market demand.

## 1. Introduction

Pork is one of the most traditional and popular meats consumed worldwide [1], and dry-cured meat products have been produced for many centuries, based on traditional practices including salting, drying, and smoking [2]. The processes of curing leads to changes in chemical composition, water activity, pH, color, and flavor of the meat [3,4,5]. In fact, the physicochemical (lipolytic and proteolytic changes, dehydration, etc.) and oxidative reactions (mainly lipid and protein oxidation) are responsible for the most important properties of the final product, as they influence the typical characteristics of dry-cured products [6,7,8,9]. However, there is a growing concern about the ways in which meat products are produced, and the consumer’s concern for the purchase of healthier products is growing also. This perception is associated not only with how meat is produced but also with how is processed by the meat industry [10]. Therefore, it is important to study the changes in the characteristics of cured meat products, since the conditions of the different processing stages exert an enormous influence on their final quality.

On the other hand, consumers demand very high-quality products, which increases the tendency of the meat industry to market products identified with quality marks, or that derive from certain animal breeds with a differentiated quality. In this sense, Bísaro pork (a local Celta breed raised in the northern area of Portugal), has been becoming more and more attractive to consumers due to its excellent meat quality [11]. Although some researchers studied the carcass characteristics [12,13] and meat quality [14], the composition, the main processes, quality of the dry-cured meat products from this breed have not been described in detail [12,15]. The dry-cured shoulder is a cured meat product from the foreleg of the pig which is cut at the shoulder blade–humerus joint, following very similar processes to those used in the production of dry-cured ham. The dry-cured shoulder is not as popular as dry-cured ham, and the information available is even scarcer. However, considering the acceptance of this product from other breeds of 62 pigs [16], and the shorter processing time compared to ham, this results in a lower price. Although there are some studies of dry-cured shoulders, mainly of Iberian pork, it is important to highlight that to date there are no studies or knowledge on the processing of dry-cured shoulder of the Bísaro breed or its quality. Consequently, a detailed characterization of dry-cured shoulders from Bísaro pigs would be interesting as a product characterization for both the meat industry and consumers. In addition, it would be necessary information to incorporate in the specification of a possible Protected Geographical Indication (PGI) brand. Therefore, the aim of the present research was to study the impact of the curing process on the chemical composition of Bísaro shoulders and to evaluate their sensory quality. The study also intended to provide quality information of a processed product at a cheaper price than ham, and to valorize one more meat product from a rare and autochthonous pork breed such as the Bísaro, which presents excellent meat characteristics for the production of meat products.

## 2. Materials and Methods

### 2.1. Dry-Cured Bísaro Shoulder

**Figure 1:**
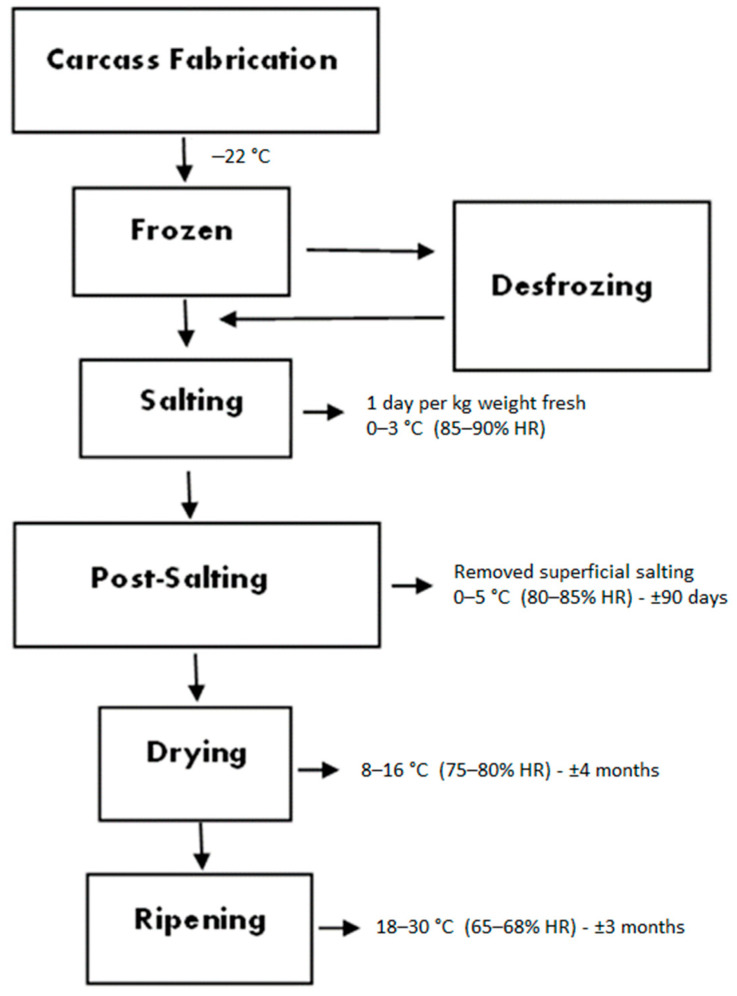
Flowchart of dry-cured Bísaro shoulder processing.

Thirty-eight animals of the Bísaro breed (*Sus scrofa*) reared in the extensive production system of a farm (Bísaro Salsicharia Tradicional^®^) from north-eastern Portugal (Gimonde, Bragança, Portugal) were randomly selected for this experiment. The animals were slaughtered when they reached 12 months of age, at approximately 120 kg live weight and 90 kg carcass weight. The slaughter procedure and carcass preparation were previously described by Álvarez-Rodríguez and Teixeira [12]. All animals were cared for and slaughtered in compliance with the welfare regulations and respecting EU Council Regulation (EC) No. 1099/2009 [17]. After the carcass refrigeration period (for 24 h between 0 and 4 °C), one shoulder from each carcass was taken (19 for raw shoulder and 19 for dry-cured shoulder), and frozen at −22 °C for 1 month. The defrosting process is carried out in a refrigeration chamber intended for this purpose, at a temperature between 0 and 5 °C. Before freezing, each piece was cleaned of part of the muscle, fat, and skin to obtain the desired form. The shoulders were processed and cured for 12 months in the “Bísaro-Salsicharia Tradicional, Lda” company. Curing and drying were carried out through several organized stages, including salting, post-salting (stabilization), drying, and ripening. For the salting period, shoulders were rubbed with salt and kept in piles of salt for 1 day per kg of fresh weight. No nitrite was added during processing. Chamber temperature for the salting period was 0–3 °C and relative humidity was 85–90%. At the end of the salting stage, superficial salt was removed from the shoulders using pressurized warm water. During the post-salting step, the shoulders were kept for 90 days at 0–5 °C and at a relative humidity of 80–85%. After achieving stabilization, the shoulders were moved to a chamber (drying step) where the temperature was gradually increased from 8 to 16 °C and the relative humidity was dropped to 75–80% (for 4 months). The shoulders were moved to another chamber (ripening step) where the temperature was gradually increased from 16 to 30 °C and at the relative humidity of 65–68% (for 3 months). A flowchart of dry-cured Bísaro shoulder processing is presented in Figure 1.

### 2.2. Chemical Composition and Physicochemical Analysis

Chemical composition (in terms of moisture, ashes, fat, and protein) of the raw and dry-cured shoulders were analyzed using established protocols. Three repetitions were carried out per variable studied (n = 3). The determination of moisture was performed according to the Portuguese standard [18]. A 3g of sample was added to 5 mL of ethanol (96% *v*/*v*). After that, samples were dried in a drying oven (Raypa DO-150, Barcelona, Spain) for 24 h at 103 ± 2 °C. Ashes were assessed according to the Portuguese standard [19]. To 3–5 g of sample, we added 1 mL of magnesium acetate (15% *w*/*v*) in crucibles. After that, the samples were subjected to 550 °C ± 25 °C during 5–6 h in a muffle furnace (Vulcan BOX Furnace Model 3-550, Yucaipa, CA, USA). Protein determination was carried out following the Portuguese standard [20] using the Kjeldahl Sampler System (K370, Flawil, Switzerland) and Digest System (K-437, Flawil, Switzerland). Two grams of sample were put in mineralization tubes with two catalyst tablets and 25 mL of sulfuric acid (97%). After mineralization completion, the distillation procedure was carried out. Finally, the distillate was titrated with hydrochloric acid solution and the required volume was recorded. All parameters were expressed in percent (g/100 g of product).

The measurement of pH was performed according to the Portuguese standard [21], using a portable potentiometer (Crison 507 pH-meter, Crison-instruments, Barcelona, Spain) equipped with a specific electrode penetrator (HI 99,163—HANNA), and calibrated with standard buffers with the following pH 4.01–7.02. Water activity (a_w_) was determined using a water activity probe (HygroPalmAw1 rotronic 8303, Bassersdorf, Switzerland) according to AOAC [22]. Meat color was estimated on the shoulder using a lightness (L*), red-greenness (a*), and yellow-blueness (b*) system with a colorimeter (Lovibond RT Series Model SP62, Tintometer Inc., Sarasota, FL, USA). This system of color was described with the coordinates L*, a*, and b* [22].

### 2.3. Fatty Acid Analysis

Fatty acid samples were analyzed in the Carcass and Meat Quality Laboratory of ESA—IPB. Two repetitions were carried out per variable studied (n = 2). The total lipids were extracted from 25 g of meat sample according to the Folch procedure [23]. Fifty mg of fat were used to determine the fatty acid profile. The fatty acids were transesterified according to the method described by Domínguez et al. [19]; a total of 4 mL of a sodium methoxide solution were added, vortexed every 5 min for 15 min at room temperature, then 4 mL of H_2_SO_4_ solution (in methanol at 50%) were and the solution was vortexed briefly. Posteriorly, 2 mL of distilled water were added, and the solution was vortexed again. The organic phase (with the methyl esters of fatty acids) was extracted with 2.35 mL of hexane. The fatty acid methyl esters separation and quantification were performed using a gas chromatograph (GC-Shimadzu 2010Plus; Shimadzu Corporation, Kyoto, Japan) provided, along with a flame ionization detector and an automatic sample injector AOC-20i and using a Supelco SP TM-2560-fused silica capillary column (100 m length, 0.25 mm i.d., 0.2 µm film thickness). The fatty acid contents were calculated using chromatogram peak areas and were expressed as g per 100 g of total fatty acid methyl esters. In addition, the percentage of saturated fatty acids (ΣSFA), monounsaturated fatty acids (ΣMUFA), polyunsaturated fatty acids (ΣPUFA), and the ratio PUFA n-6/n-3 and Σtrans were calculated according to Vieira et al. [24].

To access the lipid quality, the index of atherogenicity (IA) and the index of thrombogenicity (IT) were calculated according to Ulbricht and Southgate [25]:

(1) $$\mathrm{IA}=\frac{C12:0+4\;X\;C14:0+C160}{\Sigma \mathrm{MUFA}+\Sigma \mathrm{PUFA}}$$

(2) $$\mathrm{IT}=\frac{C14:0+C16:0+C18:0}{0.5\times \Sigma \mathrm{MUFA}+0.5\times \Sigma \mathrm{PUFAn}-6+3\times \Sigma \mathrm{PUFAn}-3+\frac{\mathrm{PUFA}\;n-3\;}{\mathrm{PUFA}\;n-6}}$$

### 2.4. Sensory Evaluation

Samples of the dry-cured Bisaro shoulders were evaluated by a trained panel. The following sensory attributes were evaluated: color, color fat, marbling, shine, aroma intensity, meat aroma, rancid aroma, acid aroma, sweet aroma, cured aroma, flavor intensity, flavor persistence, meat flavor, cured flavor, rancid flavor, salt flavor, sweet flavor, acid flavor, toughness, fibrousness, adhesiveness, and juiciness. This panel (made up of eleven elements) was created after the recruitment, selection, and training phases for the analysis of meat and meat products in accordance with the Portuguese Standard [26]. The assessors of the panel were given specific training that allowed them to be prepared to evaluate the products of the study. The whole process was conducted in the Sensory Analysis Laboratory at the Polytechnic Institute of Bragança. The conditions of the test room where the evaluation took place followed standard guidelines [27]. The temperature was maintained between 20 and 22 °C and the relative humidity was between 50 and 55%. The light in the room was white and each booth had a white light on to facilitate evaluation. Water was given to the panelists to clean the palate and remove residual flavors at the beginning of the session and between samples. Considering shoulders, the samples were divided into 1.5 mm thick slices by cutting with an industrial machine. They were wrapped in aluminum foil, placed at room temperature, and evaluated. A structured but unnumbered scale of 10 cm was used, in which the extremes represent the minimum (not very intense) and the maximum (very intense). The methodology used was that described by the Portuguese Standards [26].

### 2.5. Statistical Analysis

Data were analyzed using the statistical package JMP^®^ Pro 16.0.0 by 2021 SAS Institute Inc.© (Cary, NC, USA). Experimental data were reported as mean values and standard error of the mean. Analysis of variance (one-way ANOVA) for shoulder characterization was performed using the same software. The statistical differences were defined as *p* < 0.05.

## 3. Results and Discussion

### 3.1. Chemical Composition and Physicochemical Characteristics

The results of the chemical composition of the dry-cured Bísaro shoulder are listed in Table 1. Meat composition (in terms of moisture, protein, fat, and ashes) was similar to those reported by other authors [28,29,30] for cured products. In this sense, dry-cured lacón is a very similar meat product, made with the same carcass piece and processed with similar steps. A slightly higher protein value was observed in the current experiment than those reported by Veiga et al. [31] for raw lacón (18.05%) and dry-cured lacón (24.72%). In addition, in comparison with our findings, these authors reported a higher ash content in raw pork foreleg (5.07%) and very similar values at the end of the lacón process (8.45%), while lower fat values and higher moisture values were reported. In a more recent study, other authors reported similar values of protein (33.28%) and fat (10%), and higher amounts of moisture in cured lacón [32]. In this case, although the lacón has the same Bísaro processing steps, drying and ripening processes in dry-cured Bísaro shoulders are longer (>7 months) than in lacón (about 3–6 months), and this explains the higher moisture in lacón than in our product.

On the other hand, several authors studied and characterized dry-cured shoulders, mainly derived from Iberian pigs. Recent investigations reported that dry-cured shoulders from different Iberian genetic lines and crossbreeding presented slightly lower fat (6–8%), chlorides (2.9–3.9%), and protein (28–30%) content [33,34] than those reported by us. In another study, the lipids (7–11.5%), proteins (30–36%), and salt content (4–6%) in dry-cured Iberian shoulders [35] completely agreed with our results, which are in line with the findings reported by others [36]. Regarding moisture, some of the studies reported values (45%) [35,36] similar to those found by us, while others reported higher values (>50%) [33,34].

Differences in the pig breed, pig diet (it has an enormous influence on the fat deposition), and shoulder-processing steps and conditions (mainly time, temperature, and relative humidity) may explain these discrepancies among studies. In fact, part of these studies evidenced that genetic lines [34,37] and diets [35] could exert a great influence on the dry-cured shoulders composition. Thus, our specific processing conditions and the use of Bísaro pigs explain the low variations in comparison with the other studies discussed in the previous paragraphs.

As shown in Table 1, the curing process had a significant effect on all variables studied (*p* < 0.001). Regarding proximate composition, moisture decreased in the dry-cured shoulder, but protein, fat, and ash increased (*p* < 0.001). Those results are comparable to the values found by Teixeira et al. [38] in cured goat and sheep legs. The increases in protein (19.6 to 32.19%), fat (7.45 to 12.14%) and ash (2.21 to 8.50%) contents in the dry-cured shoulder are evidence of the effect of salting and drying during the curing process. As expected, during processing, the moisture content which reduced from 73.02% in raw meat to 44.71% at the end of drying pointed that it is caused by dehydration that occurs during the salting-drying stage [39].

The sodium chloride content of the dry-cured Bísaro shoulder, in line with the trend observed in the ash content, also increased, mainly with salting (NaCl addition), and post-salting (homogeneous salt distribution; [40]) stages, but also during the dry-ripening steps (water release and salt concentration). The final dry-cured shoulder had a chloride content of 4.12 ± 1.07% and was nitrite-free. The values obtained in Bísaro pork shoulders (4.12%) were very similar to those obtained by Reina et al. [36] and Caballero et al. [34] in Iberian shoulders, but lower than those observed by other authors, also in Iberian dry-cured shoulders [33,35], pork lacón [41], and Celta pig ham [42].

The increase in protein, fat, and ash content has been justified by the dehydration phenomena, which resulted in an increase in dry matter, mainly constituted by protein, fat, and ash. Thus, and in agreement with other studies, except for moisture, the rest of the parameters of chemical composition (expressed in g/100 g of fresh product), including chloride contents, increase with the processing steps [42,43,44]. This fact is more evident in the dry-ripening step, in which a greater release of water is produced and results in a higher concentration of the rest of the components.

On the other hand, as shown in Table 1, a_w_ and pH were affected by the curing process. In the case of water activity (a_w_) values, they decreased during the processing period due to the simultaneous decrease in moisture content and increase in salt amount. This fact agrees with those reported by several authors [42,43,44]. In our study, as expected during the curing process, the a_w_ values decreased (*p* < 0.001) until the final product was obtained. The initial a_w_ values (0.95) were slightly lower than the values obtained by other authors [39,42,44] in raw meat. In the final product, our results agree with those obtained by other authors in dry-cured shoulders [33,34,45] and other similar products, such as dry-cured ham [42,46] or dry-cured lacón [4,47]. In all these cases, a_w_ ranged between 0.85 and 0.90.

The initial pH value (5.54) was in the typical range of pH values in raw meat suitable for manufacturing and processing [42,46,47], and similar to those reported in Bísaro pork meat [48,49]. The final dry-cured shoulder had a pH value of 5.88, which demonstrated that the curing process produces a significant increase in pH (*p* < 0.001). Overall, the final pH values agree with the findings obtained by other authors for other cured products such as goat shoulder [50], Iberian pork shoulder [35,36], foal cecina [44,51], dry-cured lacón [32,47], ham [42], and Kazakh dry-cured beef [39], while a lower pH value was observed in dry-cured loin [30] and Iberian pork shoulders [34]. The significant pH increase can be attributed to the salting process, which increases sodium chloride content and consequently reduces the microbial load [52]. This also produces microbial inhibition and a lower drop in pH. Additionally, the pH increase in the Bísaro dry-cured shoulder during manufacture processing steps could be also explained by the release of alkaline compounds, such as low-weight nitrogen molecules and ammonia during proteolysis phenomena, which is well known that they produce a significant increase in pH values in dry-cured products [41,42,43]. These findings are consistent with the results reported for dry-cured bacon [53], and also from Celta pig ham [42]. In contrast, other authors did not report pH increases during the process [39,43,51], which can be related to the proteolysis intensity and other phenomena that produce a buffer effect. These differences can be explained by the different maturation times and the dry-ripening conditions required for the elaboration of different meat products.

As a general conclusion, despite the pH increase, the results after the curing process are below the critical value for meat products (<6.2). Moreover, the low moisture, pH, and a_w_ values obtained in the current experiment confirm the stability and safety of the dry-cured Bísaro shoulders. This is positive, as there is no risk of microbiological growth as has been found as well by Caballero et al. [35] in dry-cured Iberian shoulders from pigs feeding with different diets.

On the other hand, it is well known that the color of meat and meat products have great relevance in the consumer’s perception of meat quality [8,9,50]. Changes in instrumental color parameters (CIE L*a*b*) of Bísaro raw and dry-cured shoulder are shown in Table 1. The values observed in this study for Lightness (L*), red-greenness (a*), and yellow-blueness (b*) for raw foreleg were consistent with the results obtained by previous authors in Bísaro pork meat [48,49]. In a similar way, the dry-cured Bísaro shoulder also presented similar values for the color parameters to those obtained in other dry-cured products, such as foal cecina [44], Celta ham [42,46], Celta lacón [47], and Iberian shoulders [33,34,35,54,55]. This is important, especially considering that the dry-cured shoulders in this study were cured without the addition of nitrites (vital additive for the color stabilization), which indicates that a product with excellent color characteristics can be obtained from Bísaro pigs without the addition of this additive, thus producing a safer product.

During the manufacturing process, a significant decrease in all color parameters was observed (*p* < 0.001). The same behavior was observed by several authors, who reported a constant and progressive decrease of color parameters during the dry-ripening process of different meat products [42,43,44]. Certain studies suggest that drying time, and consequently water content, can also affect the color of these products [51], due to L* being related to the thin aqueous layer of the meat product [42]. Some authors found a positive and significant correlation between moisture content and L* values [56,57]; thus, there is a clear influence of dehydration in the values of this parameter. These also agree with the results obtained in foal cecina [44] and Celta ham [42], where the authors found a significant correlation between moisture and all color parameters (L*, a*, and b*), which coincides with our findings. Similarly, the water release produces pigments raised, such as myoglobin, which explains the decrease of L* and a* values during dry-ripening stages [43,58], while changes in myoglobin oxidative state can also be related to the decrease of both L* and a* parameters [8,9]. Also, the pH can influence the color parameters, and a negative correlation was found between pH and color parameters [42,59], which perfectly agrees with our results (after dry-curing process, higher pH produces lower color parameters) and partially explains the color changes during the dry-ripening process of Bísaro shoulders.

### 3.2. Fatty Acid Profile

The fatty acid profile of the raw and dry-cured Bísaro shoulder is shown in Table 2. In both cases, the most abundant saturated fatty acids (SFA) were palmitic (C16:0) and stearic (C18:0) acids, in monounsaturated fatty acids (MUFA) fraction were oleic (C18:1n-9) and palmitoleic (C16:1n-7) acids, and in polyunsaturated fatty acids (PUFA) fraction, linoleic (C18:2n-6), arachidonic (C20:4n-6) and linolenic (C18:3n-3) acids were the most representative. According to the individual fatty acids content, the highest amounts were observed for the C18:1n-9 (44–47%), followed by C16:0 (~24%) and C18:0 and C18:2n-6 with similar amounts (~10% each). Therefore, the sum of these 4 fatty acids represents about 90% of the total fatty acids of Bísaro pork shoulders. Taking this into account, in both raw and cured Bísaro shoulder, the most abundant fatty acids were MUFA, followed by SFA, and PUFA. These results agree with the typical fatty acid composition of pork and coincide with the fatty acid profile describe previously in Bísaro meat [14] and also in the Celta pigs [60,61,62,63], which belong to the same genetic line as the Bísaro pigs. Despite the fact that there are many factors that affect the fatty acid composition of the pork, including diet, breed and genetic lines, rearing system, carcass localization, etc., it is known that the profile that we have just discussed is the typical fatty profile of pig meat. In this regard, several authors reported the same trend in other similar cured meat products, including hams [36,64], Celta lacón [63], and Iberian dry-cured shoulders [33,36,54,55].

The rearing of Bísaro pigs in extensive systems allows them to consume products rich in oleic acid, such as chestnuts and acorns. This may be an explanation for the high content of MUFA in these pigs. Additionally, it must be taken into account that pig metabolism transforms excess energy from the diet through de novo synthesis into the form of saturated and monounsaturated fatty acids [61]. This is due to carbohydrates serving as substrate for the synthesis of C16:0, which are enzymatically elongated and desaturated to form C16:1n-7, C18:0, and C18:1n-9. In fact, de novo synthesis of the non-essential fatty acids, which include C16:0, C16:1n-7, C18:0, and C18:1n-9 represented more than 90% of total deposited fatty acids [63]. Moreover, the activity of the enzymes involved in this synthesis (mainly elongases and stearoyl-CoA and Δ^9^-desaturases) are modulated for other diet factors, including the dietary linoleic fatty acid, vitamin A and protein content [61]. With this in mind, both mechanisms (direct diet deposition of fatty acids and de novo synthesis) can explain the MUFA and SFA content in Bísaro shoulders, and more specifically the contents of C16:0, C18:0, and C18:1n-9.

Similar to our results, Iberian pork has been shown to have a lower SFA and higher MUFA content than other meats, which is explained by the high proportion of oleic acid in the acorns eaten by the pigs during fattening [35]. This can be an important nutritional aspect, since MUFA reduces cardiovascular risk factors [65]. Moreover, MUFA reduces plasma LDL cholesterol levels without impairing the anti-atherogenic properties of HDL cholesterol lipoproteins [66].

As aforementioned, among all parameters which can affect the fatty acid composition, diet is vital, since pigs are monogastric and have no enzymatic systems to synthetize PUFA fatty acids [61]. Thus, all PUFA are elongated and desaturated from the essential linoleic (n-6 fatty acids) and linolenic (n-3 fatty acids) fatty acids, which should be supplied in the pig diet. With this in mind and taking into account that concentrates presented high amounts of C18:2n-6 and low amounts of C18:3n-3, it is expected the amounts of both fatty acids obtained in the present study are representative. In fact, although the animals reared in extensive have the possibility of consuming grass, rich in linolenic acid, the amount ingested is insignificant compared to the intake of concentrate, which would explain the low concentrations of this fatty acid (C18:3n-3), and the high contents of linoleic acid found in the Bísaro pork shoulder. Moreover, it is well known that dietary fatty acids deposited in pork meat are diluted by SFA and MUFA fatty acids derived from de novo synthesis [36,61]. Therefore, the fatty acid profile found in the present study is in agreement with the characteristic fatty acid composition in pigs, and the high adipogenic activity of Bísaro pig (such as all Celta pig lines) determines high de novo synthesis and thus, high amounts of MUFA, medium amounts of SFA, and low amounts of PUFA, specifically very low n-3 PUFA content.

Furthermore, the PUFA/SFA and PUFA n-6/n-3 ratios were calculated. In relation to PUFA/SFA ratio, the current work had values of 0.39, within the lower limit recommended for healthy foods and diets [67]. Values obtained for similar products showed that the PUFA/SFA ratio of the Bísaro breed was within the values reported in several hams [66] (among 0.18 and 0.60), while in Iberian shoulders, the authors reported lower values (<0.30) for this ratio [34,35,36,37], and in Celta lacón, this value was higher (0.46–0.80) [63]. However, it is not necessarily healthy to have a high proportion of PUFA if the n-6/n-3 ratio is not balanced [66,68,69]. In our study, the PUFA n-6/n-3 ratio was 19.14 for the fresh foreleg and 16.08 for the cured Bísaro shoulder. These values are in agreement with the results reported in pig meat and meat products, which normally this ratio varies from 12 to 19 [35,37]. Our values were slightly higher than those reported by Caballero et al. [35] in dry-cured Iberian shoulder but similar to other studies in the same meat product [36,37]. In any case, the value of this ratio in our study exceeds the internationally recommended values for a healthy and balanced diet, which are 4 [68,70], the optimal value being 1 [69,71,72].

In addition, the present study found that the *trans* fatty acids content in the raw and dry-cured shoulder was less than 1%, as recommended [73] and lower than the levels found in other Iberian pig products [74], including the dry-cured hams “Cebo” (0.59%) and “Bellota” (0.40%). These findings suggest that the feed consumed by free-range Bísaro pigs would contain fewer *trans* fats. Atherogenic (AI) and thrombogenic (TI) indexes correlate the amounts of certain SFA, MUFA, and PUFA of the n-3 and n-6 series. It has been proposed to indicate their role in preventing or promoting pathological phenomena in humans, such as atheromas and/or the formation of thrombi [25]. The values obtained in the current experiment for both indexes had similar results to other transformed products [75,76]. The values obtained were low in each case and were in agreement with recommendations made by previous authors [77]. These data contribute to the healthy character of the dry-cured shoulder of Bísaro pigs. 

According to the results displayed in Table 2, at the end of the cured process, the predominant SFA was palmitic fatty acid (C16:0) with 24.34%, representing about 65.43% of total intramuscular SFA, followed by stearic acid (C18:0) with 10.70%, representing about 28.76% of total intramuscular SFA. No significant differences were found in total SFA (*p* > 0.05). However, both myristic acid (C14:0) and heptadecanoic acid (C17:0) were increased after the curing process (*p* < 0.001), which is consistent with what is reported by Delgado et al. [78]. Regarding MUFA and PUFA, significant differences were found. MUFA content increased with the curing process. Within MUFA content, there was a significant (*p* < 0.01) increment in the final cured product, with 51.31% of total fatty acids. This value is related to the increase (*p* < 0.001) in oleic acid (C18:1n-9), which represents about 91.70% of the total intramuscular MUFA in the dry-cured shoulder. Conversely, the PUFA content decreased (*p* < 0.001) in the final cured product, with 11.50% of the total fatty acids in the dry-cured shoulder compared to 14.45% in the raw shoulder. These values are mainly related to the decrease in linoleic acid (C18:2n-6), which represents about 82.96% of the total intramuscular PUFA, but also with the lower values of arachidonic (C20:4n-6), and linolenic (C18:3n-3) acids. Similar changes in PUFA content and linoleic acid were observed in dry-cured hams [78]. Due to enzymatic hydrolysis, a higher SFA percentage was expected at the end of the curing process. In contrast, it is expected that the proportion of PUFA decreased during the dry-ripening stages. This is due to these fatty acids being highly susceptible to suffering oxidative degradation, and they are converted into other intermediate and secondary molecules [8]. In fact, it has been reported that PUFA (due to high unsaturation) are much more sensitive to oxidative reactions than SFA or MUFA [8]. Moreover, no significant differences were found in both samples (*p* > 0.05) for *trans* fatty acids. As *trans* fatty acids did not increase significantly during the curing process, the consumers can be sure that the final product has lower contents of these harmful fatty acids. The n-6/n-3 ratio decreased significantly (*p* < 0.01) in the dry-cured shoulder which is consistent with previous findings in similar dry-cured products [39] and is related to the greater degradation of the n-6 series fatty acids (C18:2n-6 and C20:4n-6) compared to the n-3 series acids (C18:3n-3, C20:3n-3, and C20:5n-3). For the IA, a significant increase (*p* < 0.05) occurred during the dry-curing process (0.45 in fresh meat shoulder and 0.47 in dry-cured shoulder). For IT, there was also an increase, but it has not been significant (*p* > 0.05). An aspect that should be highlighted is that the entire curing process did not induce considerable changes in the IT and IA (despite a significant increase related to minimal variations of certain individual fatty acids). This observation is important from a health point of view, as these results demonstrate that cured products do not contribute more to the potential development of cardiovascular comorbidities compared to raw meat.

### 3.3. Sensory Characteristics

**Figure 2:**
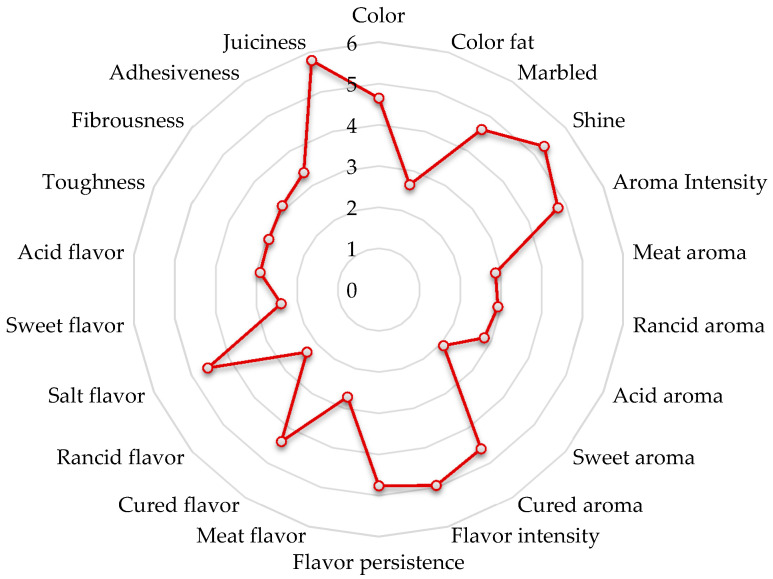
Sensory attributes of dry-cured Bísaro shoulder.

Dry-cured products are highly valued for their sensory quality, which depends on factors such as processing conditions, the animal’s genetic background, and the rearing method [79]. The mean scores obtained from the sensory analysis of dry-cured Bísaro shoulders are reported in Figure 2. Overall, 22 sensory attributes were assessed by the panelists in these types of meat products.

In this sensory analysis the juiciness, marbled, shine, aroma intensity, cured aroma, flavor intensity, flavor persistence, cured flavor, and salt flavor were those attributes reporting the highest scores. In contrast, those sensory attributes reporting the lowest values were color fat, meat aroma, rancid aroma, acid aroma, sweet aroma, meat flavor, rancid flavor, and sweet flavor (Figure 2).

The dry-cured Bísaro shoulder marbled attribute scored an average score of 4.62. For this same attribute, values between 2.37 and 5.65 were obtained for dry-cured shoulders involving different genotypes of Iberian pig [34]. Lower mean values were obtained for the dry-cured shoulder of Iberian pork from the Retinto and Torbiscal breeds (3.4–3.7) [79] and sliced Iberian dry-cured shoulder (3.3–4.5) [54]. The cured flavor attribute had an average score of 4.38 for the dry-cured shoulder of the Bísaro pig, which is in line with other studies. Similar values were also obtained for the same type of product from the Iberian pig [79], while higher values of this parameter were observed by Caballero et al. [34].

For the rancid flavor attribute, an average score of 2.31 was obtained. Lower values were also obtained by other authors for this attribute by studying the dry-cured shoulders of Iberian pork [79]. In contrast, much higher values were obtained in Iberian pigs (crossed between Retinto and Torbiscal) [34].

Concerning salt flavor attribute, the dry-cured shoulder of Bísaro pig reported average values of 4.57, being values lower than those obtained by other authors for the same type of product [34,79].

Based on the general results of the sensory analysis, it can be concluded that the panelists had positive remarks on the product.

## 4. Conclusions

The curing process caused changes in the chemical and physicochemical composition of the raw shoulder. Also, changes in the lipid profile were observed. Regarding the fatty acid profile, dry-cured Bísaro shoulders were characterized by a low proportion of SFA and a high percentage of MUFA, mainly the oleic acid. Furthermore, our study reported values within those recommended for *trans* fatty acids content, SFA/PUFA ratio, and AI and TI indexes in dry-cured Bísaro pork shoulders. From a sensory point of view, the panel of tasters positively evaluated the dry-cured shoulder. These findings are of great importance for the industry as well as for consumers, as the production of the dry-cured shoulder is cheaper, partly due to the shorter processing time. The dry-cured Bísaro shoulder is a meat product with an optimal chemical and physicochemical composition, compared to other cured products commonly consumed and it would therefore be interesting to promote its production and consumption. As a general conclusion, from the Bísaro foreleg, it is feasible to produce a high-quality nitrite-free meat product with good nutritional and physicochemical characteristics. However, future studies must be carried out, including microbiological analyzes and consumer acceptability studies, in order to guarantee the success of the product in the market.

## Figures and Tables

**Table 1:** Chemical composition and physicochemical characteristic (mean ± standard error) of raw and dry-cured Bísaro shoulder.

	Raw Shoulder	Dry-Cured Shoulder	Significance
Chemical composition (g/100 g)			
Moisture	73.02 ± 0.56	44.71 ± 0.53	***
Protein	19.60 ± 0.35	32.19 ± 0.37	***
Ashes	2.21 ± 0.15	8.50 ± 0.17	***
Fat	7.45 ± 1.98	12.14 ± 2.06	***
Chloride	-	4.12 ± 0.82	-
a_w_	0.95 ± 0.002	0.86 ± 0.002	***
pH	5.54 ± 0.03	5.88 ± 0.03	***
Color parameters			
L*	36.71 ± 0.58	32.59 ± 0.60	***
a*	19.34 ± 0.56	10.93 ± 0.55	***
b*	9.15 ± 0.34	6.51 ± 0.34	***

*** *p* < 0.001.

**Table 2:** Fatty acids profile (mean ± standard error) of raw and dry-cured Bísaro shoulder.

Fatty Acids	Raw Shoulder	Dry-Cured Shoulder	Significance
C14:0	1.09 ± 0.03	1.32 ± 0.02	***
C16:0	24.12 ± 0.30	24.34 ± 0.19	ns
C16:1n-7	2.79 ± 0.10	2.95 ± 0.06	ns
C17:0	0.25 ± 0.01	0.30 ± 0.01	***
C17:1n-7	0.32 ± 0.01	0.23 ± 0.01	***
C18:0	10.91 ± 0.15	10.70 ± 0.10	ns
C18:1n-9	44.54 ± 0.58	47.04 ± 0.37	***
9t-C18:1	0.24 ± 0.01	0.26 ± 0.01	ns
C18:2n-6	11.60 ± 0.56	9.54 ± 0.35	**
C18:3n-3	0.53 ± 0.03	0.42 ± 0.02	***
C20:1n-9	0.92 ± 0.08	0.77 ± 0.05	ns
C20:2n-6	0.49 ± 0.02	0.45 ± 0.01	ns
C20:3n-3	0.10 ± 0.005	0.09 ± 0.003	*
C20:3n-6	0.16 ± 0.01	0.12 ± 0.01	**
C20:4n-6	1.41 ± 0.07	0.66 ± 0.04	***
C20:5n-3	0.04 ± 0.01	0.13 ± 0.01	***
ΣSFA	36.72 ± 0.41	37.20 ± 0.26	ns
ΣMUFA	48.84 ± 0.65	51.31 ± 0.41	**
ΣPUFA	14.44 ± 0.65	11.50 ± 0.41	***
PUFA/SFA	0.39 ± 0.02	0.31 ± 0.01	***
PUFA n-3	0.73 ± 0.03	0.67 ± 0.02	ns
PUFA n-6	13.71 ± 0.64	10.83 ± 0.40	***
PUFA n-6/n-3	19.14 ± 0.85	16.08 ± 0.53	**
Σ*trans*	0.24 ± 0.01	0.26 ± 0.01	ns
IA index	0.45 ± 0.01	0.47 ± 0.01	*
IT index	1.08 ± 0.02	1.10 ± 0.01	ns

ns—not significant, * *p* < 0.05, ** *p* < 0.01, *** *p* < 0.001; SFA, saturated fatty acids; MUFA, monounsaturated fatty acids; PUFA, polyunsaturated fatty acids; PUFA n-6/n-3 (∑ omega-6)/(∑ omega-3); IA, index of atherogenecity; IT, ndex of thrombogenicity; only fatty acids which represented more than 0.1% are presented in the table, although all detected fatty acids were used for calculating the totals and the indices.

## Data Availability

All data were presented in the manuscript. Data can be requested from the corresponding author via email.
